# Metagenomes, Metagenome-Assembled Genomes, and Metatranscriptomes from Polychlorinated Biphenyl-Contaminated Sediment Microcosms

**DOI:** 10.1128/mra.01126-21

**Published:** 2022-06-29

**Authors:** Jessica M. Ewald, Jerald L. Schnoor, Timothy E. Mattes

**Affiliations:** a Department of Civil and Environmental Engineering, University of Iowa, Iowa City, Iowa, USA; Universidad Nacional Autónoma de México

## Abstract

We present a comprehensive data set that describes an anaerobic microbial consortium native to polychlorinated biphenyl (PCB)-contaminated sediments. Obtained from sediment microcosms incubated for 200 days, the data set includes 4 metagenomes, 4 metatranscriptomes (in duplicate), and 62 metagenome-assembled genomes and captures microbial community interactions, structure, and function relevant to anaerobic PCB biodegradation.

## ANNOUNCEMENT

Polychlorinated biphenyl (PCB)-contaminated sediments threaten human and ecological health but often harbor PCB-transforming bacteria that help detoxify sediments (1–3). We established anaerobic microcosms, as described previously (4, 5), to investigate anaerobic microbiomes native to PCB-contaminated lagoon sediments. Replicate microcosms contained sediments, collected in 2017, from two different locations (four bottles total) with variable PCB concentrations (28.04 ± 2.89 μg/mL [high-PCB microcosms [HPCBM], F4_1 and F4_2] versus 4.28 ± 1.05 μg/mL [low-PCB microcosms [LPCBM], E2_1 and E2_2]; *P* < 0.0001).

After 200 days of incubation, DNA was extracted from single slurry samples (2 mL) with a modified DNeasy PowerWater Sterivex kit protocol (6), and RNA was extracted from duplicate slurry samples (5 mL) with the RNeasy PowerSoil total RNA kit (Thermo Fisher Scientific, Waltham, MA). Contaminating DNA was removed from RNA with the Direct-zol RNA MiniPrep Plus kit (Zymo Research Corp., Irvine, CA) and the TURBO DNA-free kit (Thermo Fisher Scientific). RNA quality was confirmed using a 2100 Bioanalyzer RNA Pico assay (Agilent Technologies, Santa Clara, CA).

High-throughput DNA and RNA sequencing (4 metagenomes and 4 metatranscriptomes in duplicate) was performed at the Iowa Institute of Human Genetics (IIHG) (Iowa City, IA, USA). Indexed DNA libraries, prepared with the KAPA HyperPrep kit (Roche Sequencing and Life Science, Indianapolis, IN) using sheared DNA (average size, 550 bp), were pooled and sequenced on separate lanes of an S Prime NovaSeq 6000 flow cell (2 × 150-bp paired-end reads). RNA libraries were indexed and rRNA depleted with the stranded total RNA preparation with Ribo-Zero Plus kit (Illumina, Inc., San Diego, CA). To improve mRNA sequencing efficiency, supplemental rRNA probes developed from Methanosarcina barkeri (GenBank accession number NZ_CP009530.1) and Methanobacterium subterraneum (GenBank accession number NZ_CP017768.1) were added to further deplete methanogenic archaeal rRNA sequences. RNA was sequenced on a single Illumina NovaSeq 6000 flow cell lane (2 × 150-bp paired-end reads).

**FIG 1:**
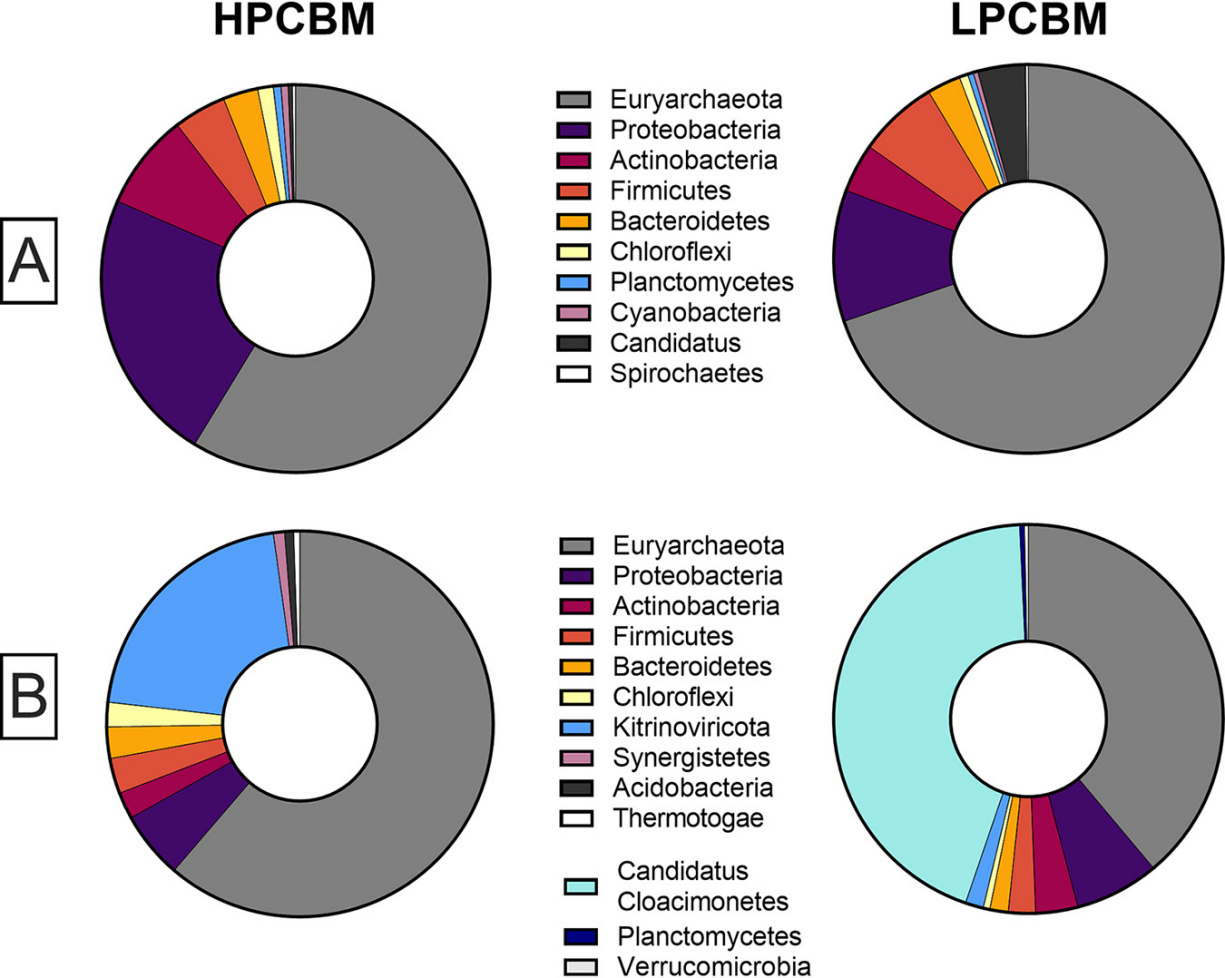
Relative abundance of reads classified at the phylum level in the HPCBM and LPCBM metagenome data sets (top 10 phyla) (A) and HPCBM and LPCBM metatranscriptome data sets (top 13 phyla) (B).

Metagenome sequencing yielded 97,279,994 (E2_1), 105,614,978 (E2_2), 102,655,932 (F4_1), and 103,672, 591 (F4_2) raw reads from each bottle. Metatranscriptome sequencing (RNA-seq) yielded 50,403,972 (E2_1 metatranscriptome-A), 60,356,358 (E2_1-B), 49,353,924 (E2_2-A), 45,023,913 (E2_2-B), 54,860,340 (F4_1-A), 47,603,523 (F4_1-B), 55,440,491 (F4_2-B), and 71,530,818 (F4_2-C) raw reads. After trimming and filtering of unassembled sequence reads with Trimmomatic (v0.39) (7), the 10 most abundant phyla in the metagenomes and the 13 most active phlya in the metatranscriptomes were determined with Kraken2 (v2.0.8) (Fig. 1).

Metagenome-assembled genomes (MAGs) were obtained by removing low-abundance k-mers from quality-filtered reads with khmer (v3.0.0) (8), coassembling paired reads into 4,213,733 contigs with Megahit (v1.2.9), and sorting contigs of >1,000 bp into bins with MetaBAT (v2.15) (9, 10). Assembly statistics were quantified with MetaQUAST (v5.0.2) (11). Bin completeness, contamination, and strain heterogeneity statistics were generated by CheckM (v1.1.3), and bin taxonomy was refined with Kraken2 (12–14) (Table 1). Among the MAGs, bin 47 (Dehalococcoides mccartyi) (Table 1), which was previously implicated in PCB dechlorination (4), is expected to harbor PCB dehalogenase genes.

**TABLE 1:** Assembly statistics and strain heterogeneity parameters for 62 high-quality MAGs

GenBank assembly accession no.	Bin no.	Taxonomy^*a*^	Completeness (%)	Contamination (%)	Strain heterogeneity (%)	No. of contigs	*N*_50_ (bp)
ASM2137325v1	10	G: Pseudomonas	95.37	4.01	40	201	34,137
ASM2137313v1	18	G: *Bacteroides*	94.62	0	0	10	455,779
ASM2137294v1	19	F: *Planctomycetaceae*	95.45	1.14	100	146	41,542
ASM2137286v1	24	G: *Proteiniphilum*	91.44	0.27	100	21	210,653
ASM2173286v1	47	G: *Dehalococcoides*	91.47	0.99	100	9	202,567
ASM2137229v1	50	G: *Bradyrhizobium*	93.06	1.85	0	56	75,318
ASM2137227v1	51	F: *Planctomycetaceae*	94.32	3.33	90.91	141	57,735
ASM2137223v1	56	C: *Spirochaetia*	91.11	2.25	50	237	13,621
ASM2137189v1	69	O: *Clostridiales*	93.15	1.96	50	347	11,116
ASM2137317v1	129	F: *Microbacteriaceae*	92.92	1.74	0	144	30,602
ASM2137318v1	132	G: *Sulfuricella*	97.03	4.53	38.46	55	93,072
ASM2137315v1	179	G: *Bacillus*	94.64	0	0	85	34,100
ASM2137311v1	182	G: *Christensenella*	96.43	1.61	50	39	90,408
ASM2137301v1	188	G: *Nitrosospira*	95.45	1.82	0	22	130,422
ASM2137302v1	193	G: Pseudomonas	94.64	0	0	64	39,167
ASM2137293v1	213	G: *Proteiniphilum*	99	0.27	0	35	233,944
ASM2137291v1	226	G: *Cloacibacilus*	90.68	1.69	100	98	29,445
ASM2137289v1	244	G: *Cloacibacilus*	100	0	0	97	37,569
ASM2137285v1	248	G: *Streptomyces*	91.67	1.85	0	700	59,520
ASM2137282v1	250	G: *Streptomyces*	97.92	3.61	40	105	29,143
ASM2137281v1	256	G: *Syntrophus*	90	4.87	25	329	16,903
ASM2137278v1	257	C: *Negativicutes*	95.76	2.26	0	74	44,539
ASM2137277v1	270	G: *Streptomyces*	93.49	4.87	25	494	11,769
ASM2137272v1	298	G: *Pelolinea*	95.25	4.73	0	200	37,672
ASM2137275v1	300	G: *Bacteroides*	95.83	1.28	20	449	15,112
ASM2137271v1	336	G: *Methanobacterium*	96.29	1.6	100	221	14,601
ASM2137269v1	341	G: *Geobacter*	97.42	3.87	0	240	41,457
ASM2137267v1	350	G: *Christensenella*	97.58	2.02	0	123	29,890
ASM2137263v1	361	G: *Syntrophobacter*	91.22	4.53	61.54	454	14,033
ASM2137261v1	367	G: *Streptomyces*	93.98	4.17	0	171	68,371
ASM2137264v1	369	G: *Clostridium*	96.77	1.69	0	105	62,815
ASM2137258v1	371	G: *Bacillus*	95.54	0	0	104	36,363
ASM2137257v1	378	O: *Rhizobiales*	96.82	3.23	33.33	214	34,208
ASM2137249v1	379	G: *Lysobacter*	97.37	2.28	0	47	126,243
ASM2137251v1	397	G: Pseudomonas	98.9	2.2	50	222	37,013
ASM2137253v1	417	F: *Enterobacteriaceae*	97.74	0.55	0	236	39,130
ASM2137255v1	426	F: *Planctomycetaceae*	96.45	2.37	75	361	19,317
ASM2137247v1	428	P: *Chloroflexi*	98.17	1.38	50	250	20,510
ASM2137243v1	430	G: *Streptomyces*	91.81	4.74	12.5	197	23,332
ASM2137242v1	451	G: Treponema	91.95	0.57	100	360	8,129
ASM2137241v1	454	G: *Desulfovibrio*	93.87	3.12	14.29	477	14,799
ASM2137237v1	460	G: *Pelosinus*	94.25	1.67	0	199	30,621
ASM2137239v1	476	G: *Desulfovibrio*	93.55	2.35	50	306	14,167
ASM2137235v1	485	G: *Rhodococcus*	97.83	2.16	36.36	281	42,192
ASM2137233v1	495	G: *Methanomassiliicoccus*	96.77	0.81	0	82	25,889
ASM2137231v1	505	G: *Methanomassiliicoccus*	97.58	1.61	100	127	26,633
ASM2137224v1	513	G: “*Candidatus* Solibacter”	100	2.24	25	368	35,769
ASM2137216v1	565	F: *Flavobacteriaceae*	92.79	4.1	50	303	10,675
ASM2137218v1	577	F: *Parachlamydiaceae*	90.03	3.04	20	380	9,230
ASM2137215v1	582	G: “*Candidatus* Protochlamydia”	91.22	4.73	14.29	402	14,309
ASM2137199v1	599	G: *Methanoregula*	99.02	0	0	43	69,042
ASM2137205v1	609	G: *Syntrophobacter*	98.48	4.53	7.69	318	20,595
ASM2137200v1	610	F: *Planctomycetaceae*	98.78	3.45	75	106	48,160
ASM2137195v1	619	G: *Bacteroides*	96.43	0.95	0	16	219,478
ASM2137196v1	625	G: *Planctomyces*	91.1	2.27	0	201	50,230
ASM2137193v1	658	O: *Enterobacterales*	95.37	3.09	0	22	313,181
ASM2137191v1	662	F: *Nitrospiraceae*	94.55	4.19	37.5	152	17,452
ASM2137187v1	687	G: *Bacteroides*	90.59	0.27	0	233	18,036
ASM2137183v1	707	G: *Methanoculleus*	91.18	0	0	88	50,994
ASM2137179v1	716	G: *Sedimentisphaera*	98.86	1.7	0	28	181,921
ASM2137185v1	736	G: *Flavobacterium*	100	2.62	0	73	198,373
ASM2137180v1	739	G: *Clostridium*	91.91	4.55	20	250	9,902

a Taxonomy was determined by the CheckM lineage workflow and further refined with Kraken2. The letters indicate the phylogenetic rank of taxonomic classification (P, phylum; C, class; F, family; O, order; G, genus).

### Data availability.

Raw data files (24 fastq files) and MAGs are available under BioProject accession number PRJNA743546. Metagenomic data are available under SRA accession numbers SRX11347095 to SRX11347098. Metatranscriptomic data are available under accession numbers SRX14430540 to SRX14430547. PCB data are available at Iowa Research Online (https://www.doi.org/10.25820/data.006156).
